# The importance of nanofiber hydrophobicity for effective fog water collection

**DOI:** 10.1039/d1ra00749a

**Published:** 2021-03-15

**Authors:** Joanna Knapczyk-Korczak, Piotr K. Szewczyk, Urszula Stachewicz

**Affiliations:** Faculty of Metals Engineering and Industrial Computer Science, AGH University of Science and Technology al. A. Mickiewicza 30 30-059 Kraków Poland ustachew@agh.edu.pl +48 12 617 52 30

## Abstract

To increase fog collection efficiency in a fiber system, controlled wetting properties are desirable. In this work, hydrophobic (PA11) and hydrophilic (PA6) polyamides were tested to verify the surface wetting effect on fog water collection rate. Highly porous fiber meshes were obtained from both polymer solutions. Randomly oriented fibers with average diameter of approximately 150 nm were observed with a scanning electron microscope (SEM). Despite the similar geometry and zeta potential of PA6 and PA11 meshes, it was shown that the hydrophobic PA11 nanofibers are more effective at water collection than hydrophilic PA6. These results indicate that wetting properties of electrospun nanofiber mesh have a significant effect on the process of draining from the mesh, as discussed in this paper. The results obtained are crucial for designing more efficient fog water collectors that include nanofibers in their construction.

## Abstract 2

This research shows the crucial role of hydrophobicity in fog water collection by polyamide (PA) nanofibers by comparing electrospun meshes from hydrophobic PA11 to those from hydrophilic PA6.

## Graphical abstract

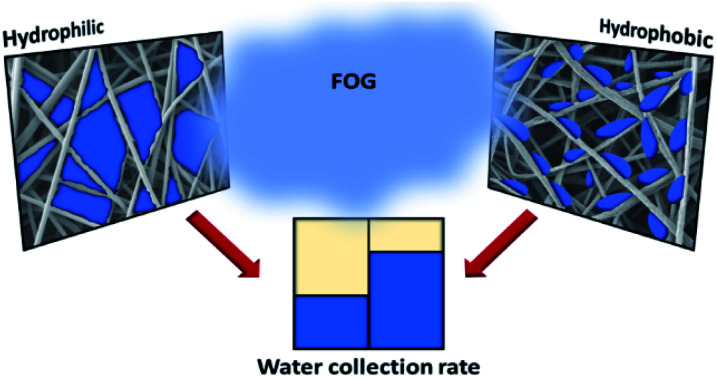

## Introduction

Water collection from fog is an effective and low-cost method of water harvesting in places where access to traditional water sources is limited.^1–3^ Commercial Fog Water Collectors (FWCs) are usually placed in foggy and windy regions, such as Chile, Morocco, Nepal and Eritrea.^4^ FWCs are specially designed meshes from polyethylene (PE) or polypropylene (PP) mounted on steel stands. They consist of a double layer of mesh with total opening range from 35% to 70%.^3,5–7^ The mechanism of water collection is relatively simple. Fog consists of small water droplets, which are moved by the wind that randomly passes through the collectors and become trapped in the mesh.^8,9^ Average diameter of water droplets can range from 17 μm at the formation stage up to 47 μm in dense fog.^10^ Such small droplets need a dedicated mesh design for satisfactory water harvesting. After impact, droplets remain on the meshes and concentrate to larger agglomerates,^11,12^ which then run down to containers under the influence of gravity and wind.^4^ Notably, for fibrous meshes, the remaining challenge is the frequent blockage of pores by growing droplets, which reduces their efficiency for collecting water.^12,13^ Often, lower water contact angle hysteresis results in a more efficient drainage system allowing faster removal of droplets, thus reducing the time of pore blockage.^14^ Water collection solutions have been also inspired by nature *e.g.* Namib Desert beetles (*Stenocara gracilipes*) where the combination of hydrophobic–hydrophilic properties is important.^15–17^ This combination of wetting properties in the form of Janus system is in the focus of research which has shown significant increase in the water collection efficiency.^18–20^ Many studies have shown the possibility and suitability of nanofibers with hydrophobic and hydrophilic properties to catch fog and water droplets from humid air.^21–24^ Nanofibers are commonly produced *via* electrospinning, where a voltage is applied between the nozzle and grounded collector.^25–28^ The electrostatic field causes elongation of the polymer solution into a jet, which moves in a spiral motion and becomes unstable due to solvent evaporation.^29,30^ Instability caused by solvent evaporation usually results in a random distribution of fibers that form the mesh. Micro and nanofibers can also be integrated into commercially available Raschel meshes, resulting in enhanced water collection.^31,32^

The mechanism of water collection in FWCs, including the draining process of deposited droplets, depends on the wetting properties of the fiber surface. For fiber meshes with randomly oriented fibers, spreading of droplets is dictated by the surface wettability, texture of the mesh and individual fibers influencing the water contact angle. Wetting properties considering roughness are described by Wenzel regime, or its theoretical modification, for hydrophilic surfaces and in a Cassie–Baxter state, or its theoretical modification, for hydrophobic surfaces.^33–35^ Therefore, the surface properties, size of fibers and fiber fraction, together with their shape and roughness, affect the wettability of a mesh, which translates to fog collection efficiency.^23^ Interestingly, a combination of hydrophobic and hydrophilic properties can be used to enhance the water collection rate^36,37^ of FWCs by changing the wetting and drainage system from the vertically placed meshes where the gravity has its effect.^38^

Another aspect in fog collection is the surface charges commonly used in electrostatic collectors.^39^ The influence of an electric field causes polarization of water molecules which, in consequence, accelerates their coalescence and growth.^40^ This approach is based on a high electric field, which imparts a net charge to the incoming droplets, and they are directed to the collector by electrostatic force.^41^ The optimization of the fibrous meshes is crucial for efficient water drainage and high-water collection rate in FWCs. Water behavior in contact with material at macro, micro and nanoscale may be drastically different.^42^

**Fig. 1:**
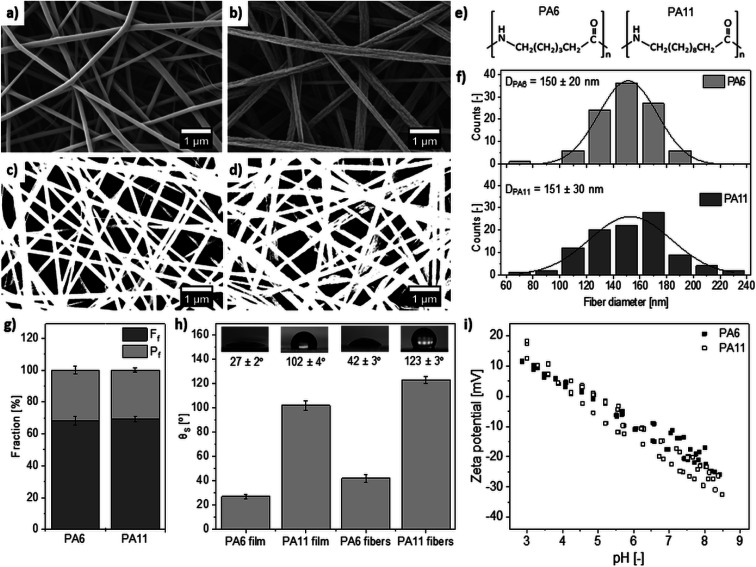
The SEM images of nanofibers: (a) PA6, (b) PA11. The representative images used in image analysis: (c) PA6, (d) PA11. (e) Structure of repeat units of PA6 and PA11. (f) The histograms of nanofiber diameter distribution. (g) The comparison of fiber *F*_f_ and pore *P*_f_ fractions in the polyamide meshes. (h) Static contact angles for fibers and films. (i) The streaming zeta potential of PA6 and PA11 meshes.

In this work we want to compare two materials with a significant difference in wetting properties, but with similar average fiber diameters and chemical structure (Fig. 1). Therefore, we investigated polyamides (PA): hydrophobic PA11 and hydrophilic PA6 electrospun nanofibers with similar diameters. The PA meshes have similar pore size and porosity, however, the difference in the number of carbon atoms in the polymer chain structure changes the wettability of PA meshes. By investigating the mechanisms of water collection for both systems and their effects on water collection efficiency, we are able to determine, which wetting properties of materials are more favorable for efficient fog water collection for nano-scale materials at very low wind conditions.

## Experimental

### Materials and sample preparation

#### Materials and electrospinning

Polyamide 6 (PA6, (C_6_H_11_NO)_*n*_, *M*_w_ = 24 000 g mol^−1^; BASF, Germany) and polyamide 11 (PA11, (C_11_H_21_NO)_*n*_, *M*_w_ = 204.31 g mol^−1^; Sigma Aldrich, USA) (see Fig. 1e) were used to prepare electrospinning solutions with concentrations of 12% and 6%, respectively. To evaporate water from the hygroscopic PA6 granules, they were dried at 40 °C until constant weight was obtained. The polymer was then dissolved in a mixture of formic and acetic acids in 1 : 1 volume ratio (CH_2_O_2_ > 98%; C_2_H_4_O_2_ > 99.5%, Avantor Performance Materials Poland S.A., Poland) at *T* = 25 °C, according to previous protocols.^23,31,43^ The PA11 was dissolved in formic acid at *T* = 60 °C. The solutions were stirred for 4 h at a constant speed of 500 rpm for PA6 and 700 rpm for PA11 (RCT basic, IKA, Germany).

PA nanofibers were produced *via* electrospinning in a chamber with environmental control (IME Technologies, The Netherlands).^44^ The electrospinning parameters are listed in Table 1. A hypodermic injection needle was used as a nozzle (hypodermic injection needle KD Fine 0.8 × 40 mm 21 G × 1 1/2′′ green, KD Medical GmbH Hospital Products, Germany). The meshes were electrospun on a rotating drum (diameter = 9 cm, length = 18 cm) spinning at 10 rpm. Fibers were electrospun onto baking paper for easier removal of the mesh from the collector. For static contact angle (*θ*_S_) measurement, the samples were deposited on glass slides. Additionally, thin films for the *θ*_S_ measurements were prepared by placing 0.1 ml of solution on a glass slide (16 × 16 mm) and spin-coating (L2001A v.3, Ossila, Sheffield, UK) at a rotation speed of 1000 rpm for 30 s. Then, films were dried in a fume cupboard for 24 h at 25 °C.

**Table 1:** Electrospinning parameters

	Flow rate [ml h^−1^]	Distance [cm]	Voltage [kV]	Temperature [°C]	Humidity [%]	Time [h]
PA6	0.1	15	16	25	40	3
PA11	0.25	12	18	25	50	3

### Mesh characterization

#### SEM and image analysis

A scanning electron microscope (SEM, Merlin Gemini II, ZEISS, Germany) was used to analyze nanofiber and film morphology. Prior to imaging, samples were coated with a 10 nm gold layer using a rotary pump sputter coater (Q150RS, Quorum Technologies, UK). The SEM imaging was performed with an accelerating voltage of 2.5 kV and current of 110 pA at a working distance of 7 mm. ImageJ software (version 1.50i, National Institutes of Health, USA) was utilized to determine nanofiber diameters and calculate fiber (*F*_f_) and pore (*P*_f_) fractions from SEM macrographs. The sum of *F*_f_ and pore *P*_f_ should reach about 100%. The *F*_f_ supports the similar information about the meshes as the commonly used shade coefficient in FWC.^5,13^ The average nanofiber diameter was calculated from 100 measurements presented in histograms prepared using OriginPro (2018b, OriginLab, USA). The fraction of fiber and pore analysis were performed using the particle function in ImageJ based on the images showed in Fig. 1c and d. The pore size was calculated from SEM binary images prepared using the Li thresholding method in ImageJ.^45,46^ The threshold was set to 0–90 for *P*_f_ and pore size calculation. The *F*_f_ was calculated from inverted binary 2D images.

### Wetting properties

#### Static contact angle

The static contact angle (*θ*_S_) was measured in the horizontal position using the sessile drop method at 25 °C and 40% RH. The deionized water (DI, Spring 5UV purification system – Hydrolab, Poland) was applied as 3 μl volume droplets. The images were taken by a camera with macro lens (EOS 700D, EF-S 60 mm f/2.8 Macro USM, Canon, Japan) 3 s after the droplets were placed. The *θ*_S_ was determined from the images using ImageJ.

#### Streaming zeta potential

A high-end electrokinetic analyzer was used for zeta potential analysis (SurPASS 3, Anton Paar, Austria). The streaming potential was measured between two meshes with dimensions 20 × 10 mm placed in the cell with the adjustable gap set to 110 μm. The pH value was controlled in acidic (5.6–3.0) and basic (5.6–8.0) ranges with pH steps of 0.3. The titrations were done by the progressive addition of 0.05 M HCl or 0.05 M NaOH to a 0.01 M KCl solution. The zeta potential measurement was repeated 4 times at every pH value with two rinse cycles performed before each test.

#### Fog collection experiment

The meshes used for fog collection (10 × 10 cm) were placed in a specially designed setup.^31,32,43^ Fog was produced by a humidifier (Beurer GmbH, Germany) at a flow rate set to 400 ml h^−1^. The humidifier output was set at 90° angle to, and 6 cm distance from, the vertically placed mesh, which led to a humidity of 95% during tests.^43^ This is a typical setup used in the laboratory conditions to simulate fog,^41,47^ which is characterized with the water droplet size of 250 nm and above.^48^ The experiments are performed in very low wind conditions, with the fog flow velocity of 0.19 m s^−1^. The water recovered from fog was collected in a beaker placed underneath the mesh and weighed every 30 min over 3 h of fog collection. The amount of water collected was obtained by normalizing the mass of water to the area, and the water collection rate was calculated by dividing the water collected by time.^14,43^ The pH of collected water was measured using the pH-metric electrode from the electrokinetic analyzer (SurPASS 3, Anton Paar, Austria).

## Results and discussion

### Material characterization

The randomly oriented electrospun polyamide meshes observed with an SEM showed similar average fiber diameters of 150 ± 20 nm and 151 ± 30 nm for PA6 and PA11, respectively (Fig. 1a and b). The distributions of fiber diameter are presented as histograms (Fig. 1f). The analysis of fiber and pore fraction based on the 2D SEM images confirmed the geometrical similarity of PA6 and PA11 meshes (Fig. 1c and d), as the *F*_f_ and *P*_f_ for the two meshes were almost identical (Fig. 1g). Due to the small fiber diameter, the PA meshes presented a closely packed structure when observed by the SEM, Fig. 1a and b, which extended to nearly 70% of the membrane area. The analysis of pore size resulted in an average value of 9800 ± 2135 nm^2^ for PA6 and 10 800 ± 2135 nm^2^ for PA11. Importantly, this 2D analysis can be extended to the 3D investigation using the dual beam microscopy based on focused ion beam (FIB) and SEM to obtain the 3D reconstructions of electrospun fibers network.^49^ The previous study using the 3D analysis based on FIB-SEM microscopy found that the porosity reached 96% for similar PA6 membranes.^50^

Importantly, the different wetting of PA6 and PA11 was confirmed by the static contact angle measurements^23,51–53^ (Fig. 1h). Contact angles measured on the films were 27 ± 2° for PA6 and 102 ± 4° for PA11. Measurements on electrospun fibers showed an increase in contact angle for both polymers to 42 ± 3° and 123 ± 3° for PA6 and PA11, respectively. A similar value for electrospun PA6 has been reported previously.^43^ Increase of contact angle was caused by geometry and roughness effects of the wetted fibers,^22,23^ which falls in line with Wenzel and Cassie Baxter models.

The chemical analyses of both polyamides have previously been investigated for electrospun PA6 (ref. 54) indicating the characteristic peaks at the wavenumber range 1000–3500 cm^−1^. The amide I and II peaks found between 1500 and 1700 cm^−1^, and at 3300 cm^−1^ and 2850–2950 cm^−1^ represent hydrogen-bonded N–H stretching and CH_2_ asymmetric and symmetric stretching. For PA11 (ref. 55) the peaks are in the wavenumber range 2600–3600 cm^−1^, where N–H stretching amide I is observed at 3300 cm^−1^. PA6 nanofibers were also characterized in term of their surface free energy showing enhanced wetting behavior in comparison to PA6 films.^53^

### Zeta potential measurement

To verify the surface charge effect of electrospun meshes on the water collection efficiency, streaming zeta potential measurements were performed. The zeta potential analysis showed a decrease in the streaming potential with an increase of pH for both types of polyamide samples (Fig. 1i). The isoelectric points for hydrophilic and hydrophobic meshes were similar and occurred at pH = 4.94 for PA6 and pH = 4.89 for PA11. Only at these pH values, does the zeta potential of the meshes equal 0 mV.^56^ Such behavior is important as the fog contains air pollution, which influences pH. A typical range of pH for fog water is 3.5–6.^3,57,58^ The pH range chosen in this study allowed prediction of the electrical potential of meshes in contact with natural fog water with known pH. The electrostatic interactions with water were the same for both PA meshes. The zeta potential measurements were performed to confirm the electrical neutrality of PA meshes and eliminate the possibility that the surface charges have an impact on the water collection efficiency for each case.

### Fog water collection

**Fig. 2:**
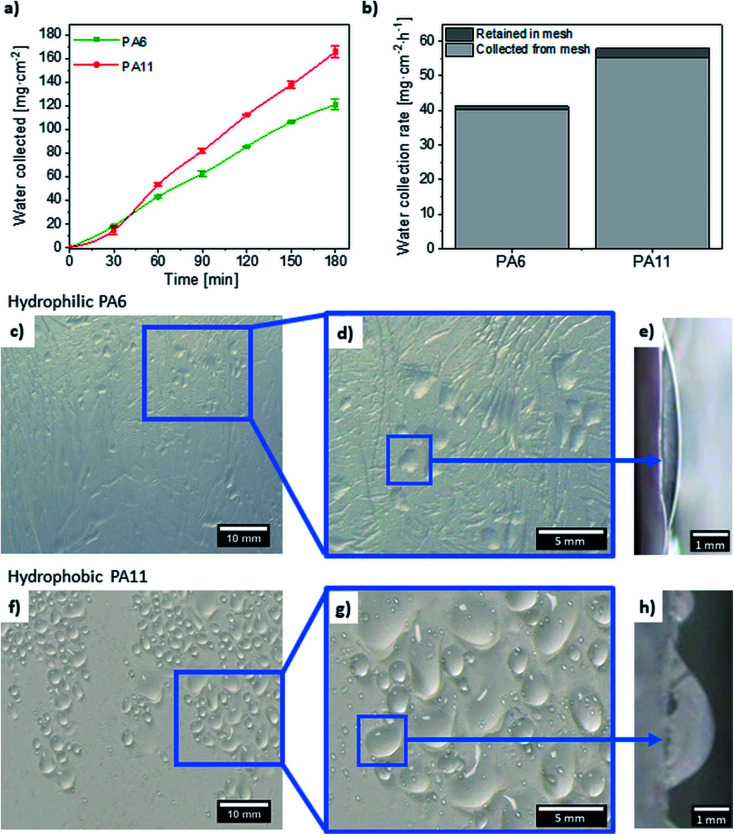
(a) Water collected by PA6 and PA11 meshes. (b) Water collection rate calculated per hour. (c–h) Front images of droplets on the nanofiber meshes after 30 minutes of fog collection; (e and g) enlarged side images of the individual droplet collected on vertical hydrophilic PA6 and hydrophobic PA11 meshes, respectively.

Water collection curves (Fig. 2a and b) for PA11 increased faster than for PA6, as reflected in water collection rate. The pH of collected water was 5.9, because the fog can absorb the CO_2_ from the air. Water collection rate including water collected from mesh and retained between fibers was 41 ± 2 mg cm^−2^ h^−1^ for PA6 and 58 ± 2 mg cm^−2^ h^−1^ for PA11. Hydrophobic PA11 mesh was more efficient at water collection than hydrophilic PA6. These results show that higher values are due to the hydrophobic material as the average fiber diameters and pore sizes were similar. However, blocking of pores by water droplets was observed for hydrophilic PA6. The observation of water droplets remaining on the mesh during fog collection (Fig. 2c and d) shows different wetting properties of the two materials investigated. Hydrophilic PA6 nanofibers allow permeation of water into empty spaces between them. Due to the hydrophobic nature of PA11, the droplets can drain more easily to the beaker due to reduction of blocking in the pores during fog droplet collection. The analysis of the SEM micrographs, fiber fractions and zeta potential results indicated similar fiber diameter and surface potential for the PA meshes. This suggests that the water collection of the two PA meshes is mainly dependent on the wetting properties of the material, which is influenced by their chemical structure. The number of carbon atoms in a molecule is greater for hydrophobic PA11 (11 at. C) than for hydrophilic PA6 (6 at. C). This is consistent with the expected increase in a given *θ*_s_ for a given polyamide with the number of carbon atoms in a single molecule of the polymer chain.^51^

The water collection rate obtained for PA6 is similar to results from a previous study for hydrophilic cellulose acetate fibrous meshes with average fiber diameter of 0.54 ± 0.16 μm, which reached 45 mg cm^−2^ h^−1^.^59^ Also, the water collection rate for hydrophobic polystyrene (PS) microfibers with average diameter of 4.80 ± 0.22 μm achieved 59 mg cm^−2^ h^−1^,^59^ which is comparable to PA11. However, the water transport from PS fibers was problematic due to high *P*_f_ (60 ± 3%). Additionally, the PS mesh had much larger pore size of 67.59 ± 8.30 μm^2^,^59^ which is three orders of magnitude higher than for PA11 mesh. The large spaces between the microfibers trap the water droplets for longer, slowing down the drainage, which decreases the water collection efficiency.^4,13^ The clogging of mesh was reduced with a thinner fiber layer, similarly to the system inspired by plants,^60^ where water collection depends on the thickness of a thin fibrous layer. The homogeneous PA11 nanofiber mesh investigated in this work allows faster water drainage from the mesh while maintaining high collection efficiency.

The shape of the water droplets shown in Fig. 2c–h clearly indicates the different wetting and drainage mechanism of PA meshes. The side view images of water droplets on the vertical meshes during the fog collection (Fig. 2e for PA6 and Fig. 2h for PA11) indicate clear variations in the contact angle hysteresis, which is approximately 3° for hydrophilic PA6 and 21° for hydrophobic PA11 on representative images. This drastic difference in wetting behavior resulted in different water removal processes for the samples measured. It is also observed in the retained water after water collection experiment, where for hydrophobic PA11 is higher than for hydrophilic PA6, see Fig. 2b. Importantly, the number of droplets collected on PA11 fibers is higher than on PA6 fibers, as indicated on the images in Fig. 2c and f. The water has not managed to flow down to the beaker as more water droplets are collected on the mesh, see Fig. 2f.

**Fig. 3:**
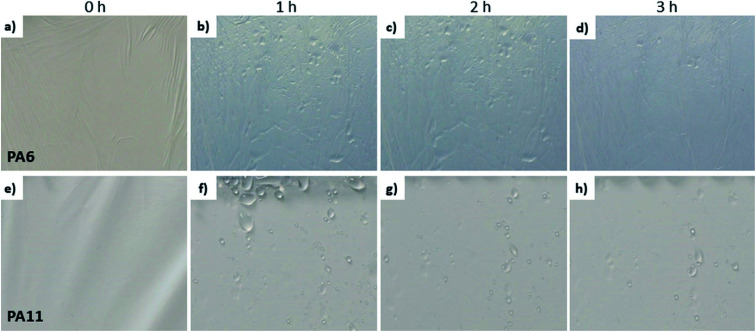
The polyamide meshes (a–d) PA6 and (e–h) PA11 before water collection, 0 h, and after 1, 2 and 3 h of fog collection.

Using polyethylene terephthalate (PET) fibers, Azad *et al.*^61^ showed a directional water droplet transport on the surface of mesh, proving the effect of surface structures on the efficiency of fog collection. The hierarchical structure of hydrophobic PVDF fibers improved the water collection efficiency of the membrane. PVDF fibers with average diameter of 650 ± 25 nm were modified to obtain nanopillars on their surface. The nanopillars were 140 ± 20 nm high and had a diameter around 150 nm, which is comparable with the PA6 and PA11 nanofiber diameters in this work. The water collection rate for just neat PVDF nanofibers and modified PVDF nanofibers with nanopillars reached 27 mg cm^−2^ h^−1^ and 81 mg cm^−2^ h^−1^, respectively. The investigated here hydrophobic PA11 mesh had a lower water collection rate than the PVDF with nanopillars, but higher than the just neat PVDF fibers with a 4 times larger diameter presented in the work of Ganesh *et al.*^62^ This suggests that solutions based on materials in the nanometric scale allow an increased fog collection rate. The nano-roughness of electrospun fibers is known to increase their hydrophobicity,^63,64^ which is crucial to obtain a faster water drainage mechanism, thus increasing water collection efficiency. The fog collection mechanism based on the hydrophobic PA11 allows a higher water collection rate to be obtained due to the faster removal of water droplets. These findings are opposite to what was observed in a previous study on microfiber meshes.^43,59^ The water capture and blockage effect can also be observed on meshes containing, not only nanofibers, but also microfibers. The PS-PA6 composites accumulated a similar amount of water as PS. The addition of PA6 nanofibers ameliorated poor drainage and the water was running off the mesh instead of clogging the pores.^43^ In Fig. 3 the photographs of water collected on PA6 meshes show a poor drainage system opposite to PA11 meshes, where the water droplets form dripping channels. However, the hydrophilicity of PA6 takes the advantage in attracting water molecules as it has been discussed in other systems.^31^ Here, we show that in terms of attracting water molecules in the electrospun mashes the main effect increasing their water collection efficiency is related to the sufficient drainage system, leaving the space for the next water droplet to be captured.

## Conclusions

The water collection properties of two meshes were measured. The hydrophobic PA11 mesh had a 40% higher fog water collection rate than the hydrophilic PA6 mesh, despite the fact that both materials had a similar value of the streaming zeta potential in the given pH range. The wetting properties of polyamides are the main factor influencing the water collected as both meshes have similar fiber diameters. The surface chemistry driven by the polymer structure and the amount of carbon in the polymer chains determines the wetting properties of polyamides. The results obtained indicate the importance of wetting properties of the material selected for mesh production and the effect of hydrophobicity at the nanoscale. The fog collection mechanism based on the hydrophobic PA11 allows a higher water collection rate to be obtained due to the faster removal of water droplets. These findings are opposite to what was observed in a previous study on microfiber meshes.^43^ Fundamental knowledge for the further development of fog collectors, including nanotechnology, is provided, as there is a growing need to seek novel approaches to the global water crisis.

## Data availability

Any additional data from this work are available from the corresponding author upon reasonable request.

## Conflicts of interest

The authors declare that they have no known competing financial interests or personal relationships that could have appeared to influence the work reported in this paper.

## Supplementary Material

## References

[cit1] Korkmaz S., Kariper I. A. (2020). Fog harvesting against water shortage. Environ. Chem. Lett..

[cit2] Qadir M., Jiménez G., Farnum R., Dodson L., Smakhtin V. (2018). Fog Water Collection: Challenges beyond Technology. Water.

[cit3] Klemm O., Schemenauer R. S., Lummerich A., Cereceda P., Marzol V., Corell D., Van Heerden J., Reinhard D., Gherezghiher T., Olivier J., Osses P., Sarsour J., Frost E., Estrela M. J., Valiente J. A., Fessehaye G. M. (2012). Fog as a fresh-water resource: overview and perspectives. Ambio.

[cit4] Fernandez D. M., Torregrosa A., Weiss-Penzias P. S., Zhang B. J., Sorensen D., Cohen R. E., McKinley G. H., Kleingartner J., Oliphant A., Bowman M. (2018). Fog Water Collection Effectiveness: Mesh Intercomparisons. Aerosol Air Qual. Res..

[cit5] Schemenauer R. S., Cereceda P. (1994). A Proposed Standard Fog Collector for Use in High-Elevation Regions. J. Appl. Meteorol..

[cit6] Swarndeep S. (2016). Design Optimisation of Fog Collector. Int. J. Innov. Sci. Eng. Technol..

[cit7] Park K. C., Chhatre S. S., Srinivasan S., Cohen R. E., McKinley G. H. (2013). Optimal design of permeable fiber network structures for fog harvesting. Langmuir.

[cit8] Schemenauer R. S., Cereceda P. (1994). The Role of Wind in Rainwater Catchment and Fog Collection. Water Int..

[cit9] Rivera J. d. D. (2011). Aerodynamic collection efficiency of fog water collectors. Atmos. Res..

[cit10] Liu Q., Wu B., Wang Z., Hao T. (2020). Fog Droplet Size Distribution and the Interaction between Fog Droplets and Fine Particles during Dense Fog in Tianjin, China. Atmosphere.

[cit11] Li C., Liu Y., Gao C., Li X., Xing Y., Zheng Y. (2019). Fog Harvesting of a Bioinspired Nanocone-Decorated 3D Fiber Network. ACS Appl. Mater. Interfaces.

[cit12] Rajaram M., Heng X., Oza M., Luo C. (2016). Enhancement of fog-collection efficiency of a Raschel mesh using surface coatings and local geometric changes. Colloids Surf., A.

[cit13] Park K. C., Chhatre S. S., Srinivasan S., Cohen R. E., McKinley G. H. (2013). Optimal design of permeable fiber network structures for fog harvesting. Langmuir.

[cit14] Lalia B. S., Anand S., Varanasi K. K., Hashaikeh R. (2013). Fog-Harvesting Potential of Lubricant-Impregnated Electrospun Nanomats. Langmuir.

[cit15] Yu Z., Zhang H., Huang J., Li S., Zhang S., Cheng Y., Mao J., Dong X., Gao S., Wang S., Chen Z., Jiang Y., Lai Y. (2021). Namib desert beetle inspired special patterned fabric with programmable and gradient wettability for efficient fog harvesting. J. Mater. Sci. Technol..

[cit16] Bhushan B. (2019). Bioinspired water collection methods to supplement water supply. Philos. Trans. R. Soc., A.

[cit17] Bhushan B. (2020). Design of water harvesting towers and projections for water collection from fog and condensation. Philos. Trans. R. Soc., A.

[cit18] Li D., Fan Y., Han G., Guo Z. (2021). Multibioinspired Janus membranes
with superwettable performance for unidirectional transportation and fog collection. Chem. Eng. J..

[cit19] Cheng L., Xu Q., Jia X., Zhang R., Bai S., Qin Y., Wang X. (2021). Anisotropic wetting properties of oblique nanowires array and their applications on water transportation and fog collection. Surf. Interfaces.

[cit20] Cao M., Xiao J., Yu C., Li K., Jiang L. (2015). Hydrophobic/Hydrophilic Cooperative Janus System for Enhancement of Fog Collection. Small.

[cit21] Gurera D., Bhushan B. (2019). Optimization of bioinspired conical surfaces for water collection from fog. J. Colloid Interface Sci..

[cit22] Bhushan B., Jung Y. C., Koch K. (2009). Micro-, nano- and hierarchical structures for superhydrophobicity, self-cleaning and low adhesion. Philos. Trans. R. Soc., A.

[cit23] Szewczyk P. K., Ura D. P., Metwally S., Knapczyk-Korczak J., Gajek M., Marzec M. M., Bernasik A., Stachewicz U. (2019). Roughness and Fiber Fraction Dominated Wetting of Electrospun Fiber-Based Porous Meshes. Polymers.

[cit24] Dong H., Wang N., Wang L., Bai H., Wu J., Zheng Y., Zhao Y., Jiang L. (2012). Bioinspired electrospun knotted microfibers for fog harvesting. ChemPhysChem.

[cit25] Garg K., Bowlin G. L. (2011). Electrospinning jets and nanofibrous structures. Biomicrofluidics.

[cit26] Huang Z. M., Zhang Y. Z., Kotaki M., Ramakrishna S. (2003). A review on polymer nanofibers by electrospinning and their applications in nanocomposites. Compos. Sci. Technol..

[cit27] Baji A., Abtahi M., Ramakrishna S. (2014). Bio-Inspired Electrospun Micro/Nanofibers with Special Wettability. J. Nanosci. Nanotechnol..

[cit28] Greiner A., Wendorff J. H. (2007). Electrospinning: a fascinating method for the preparation of ultrathin fibers. Angew. Chem., Int. Ed..

[cit29] Theron S. A., Zussman E., Yarin A. L. (2004). Experimental investigation of the governing parameters in the electrospinning of polymer solutions. Polymer.

[cit30] Hohman M. M., Shin M., Rutledge G., Brenner M. P. (2001). Electrospinning and electrically forced jets. I. Stability theory. Phys. Fluids.

[cit31] Knapczyk-Korczak J., Szewczyk P. K., Ura D. P., Berent K., Stachewicz U. (2020). Hydrophilic nano fibers in fog collectors for increased water harvesting efficiency. RSC Adv..

[cit32] Knapczyk-Korczak J., Szewczyk P. K., Ura D. P., Bailey R. J., Bilotti E., Stachewicz U. (2020). Improving water harvesting efficiency of fog collectors with electrospun random and aligned polyvinylidene fluoride (PVDF) fibers. Sustainable Mater. Technol..

[cit33] Stachewicz U., Bailey R. J., Zhang H., Stone C. A., Willis C. R., Barber A. H. (2015). Wetting Hierarchy in Oleophobic 3D Electrospun Nanofiber Networks. ACS Appl. Mater. Interfaces.

[cit34] Choi W., Tuteja A., Mabry J. M., Cohen R. E., McKinley G. H. (2009). A modified Cassie-Baxter relationship to explain contact angle hysteresis and anisotropy on non-wetting textured surfaces. J. Colloid Interface Sci..

[cit35] Wang Z., Elimelech M., Lin S. (2016). Environmental Applications of Interfacial Materials with Special Wettability. Environ. Sci. Technol..

[cit36] Zhang L., Wu J., Hedhili M. N., Yang X., Wang P. (2015). Inkjet printing for direct micropatterning of a superhydrophobic surface: toward biomimetic fog harvesting surfaces. J. Mater. Chem. A.

[cit37] White B., Sarkar A., Kietzig A. M. (2013). Fog-harvesting inspired by the stenocara beetle - an analysis of drop collection and removal from biomimetic samples with wetting contrast. Appl. Surf. Sci..

[cit38] Dorrer C., Rühe J. (2008). Mimicking the stenocara beetle – dewetting of drops from a patterned superhydrophobic surface. Langmuir.

[cit39] Cruzat D., Jerez-Hanckes C. (2018). Electrostatic fog water collection. J. Electrost..

[cit40] Gabyshev D. N., Fedorets A. A., Aktaev N. E., Klemm O., Andreev S. N. (2019). Acceleration of the condensational growth of water droplets in an external electric field. J. Aerosol Sci..

[cit41] Damak M., Varanasi K. K. (2018). Electrostatically driven fog collection using space charge injection. Sci. Adv..

[cit42] Meng S., Greenlee L. F., Shen Y. R., Wang E. (2015). Basic science of water: challenges and current status towards a molecular picture. Nano Res..

[cit43] Knapczyk-Korczak J., Ura D. P., Gajek M., Marzec M. M., Berent K., Bernasik A., Chiverton J. P., Stachewicz U. (2020). Fiber-Based Composite Meshes with Controlled Mechanical and Wetting Properties for Water Harvesting. ACS Appl. Mater. Interfaces.

[cit44] Krysiak Z. J., Gawlik M. Z., Knapczyk-Korczak J., Kaniuk L., Stachewicz U. (2020). Hierarchical composite meshes of electrospun PS microfibers with PA6 nanofibers for regenerative medicine. Materials.

[cit45] Li C. H., Lee C. K. (1993). Minimum cross entropy thresholding. Pattern Recogn..

[cit46] Stachewicz U., Szewczyk P. K., Kruk A., Barber A. H., Czyrska-Filemonowicz A. (2019). Pore shape and size dependence on cell growth into electrospun fiber scaffolds for tissue engineering: 2D and 3D analyses using SEM and FIB-SEM tomography. Mater. Sci. Eng., C.

[cit47] Hou Y., Chen Y., Xue Y., Zheng Y., Jiang L. (2012). Water collection behavior and hanging ability of bioinspired fiber. Langmuir.

[cit48] Hung D. V., Tong S., Nakano Y., Tanaka F., Hamanaka D., Uchino T. (2010). Measurements of particle size distributions produced by humidifiers operating in high humidity storage environments. Biosyst. Eng..

[cit49] Metwally S., Karbowniczek J. E., Szewczyk P. K., Marzec M. M., Gruszczyński A., Bernasik A., Stachewicz U. (2019). Single-Step Approach to Tailor Surface Chemistry and Potential on Electrospun PCL Fibers for Tissue Engineering Application. Adv. Mater. Interfaces.

[cit50] Stachewicz U., Modaresifar F., Bailey R. J., Peijs T., Barber A. H. (2012). Manufacture of void-free electrospun polymer nanofiber composites with optimized mechanical properties. ACS Appl. Mater. Interfaces.

[cit51] Fort T. , in Contact Angle, Wettability, and Adhesion, ed. F. M. Fowkes, American Chemical Society, Washington, 1964, pp. 302–309

[cit52] Moshynets O., Bardeau J.-F., Tarasyuk O., Makhno S., Cherniavska T., Dzhuzha O., Potters G., Rogalsky S. (2019). Antibiofilm Activity of Polyamide 11 Modified with Thermally Stable Polymeric Biocide Polyhexamethylene Guanidine 2-Naphtalenesulfonate. Int. J. Mol. Sci..

[cit53] Stachewicz U., Barber A. H. (2011). Enhanced wetting behavior at electrospun polyamide nanofiber surfaces. Langmuir.

[cit54] Stachewicz U., Hang F., Bailey R. J., Gupta H. S., Frogley M. D., Cinque G., Barber A. H. (2012). Recording IR spectra for individual electrospun fibers using an *in situ* AFM-synchrotron technique. Mater. Res. Soc. Symp. Proc..

[cit55] Pu X., Zha J.-W., Zhao C.-L., Gong S.-B., Gao J.-F., Li R. K. Y. (2020). Flexible PVDF/nylon-11 electrospun fibrous membranes with aligned ZnO nanowires as potential triboelectric nanogenerators. Chem. Eng. J..

[cit56] Bauman M., Košak A., Lobnik A., Petrinić I., Luxbacher T. (2013). Nanofiltration membranes modified with alkoxysilanes: surface characterization using zeta-potential. Colloids Surf., A.

[cit57] Klemm O., Tseng W.-T., Lin C.-C., Klemm K., Lin N.-H. (2015). pH Control in Fog and Rain in East Asia: Temporal Advection of Clean Air Masses to Mt. Bamboo, Taiwan. Atmosphere.

[cit58] Nieberding F., Breuer B., Braeckevelt E., Klemm O., Song Q., Zhang Y. (2018). Fog water chemical composition on ailaoshan mountain, Yunnan province, SW China. Aerosol Air Qual. Res..

[cit59] Knapczyk-Korczak J., Zhu J., Ura D. P., Szewczyk P. K., Gruszczyński A., Benker L., Agarwal S., Stachewicz U. (2021). Enhanced Water Harvesting System and Mechanical Performance from Janus Fibers with Polystyrene and Cellulose Acetate. ACS Sustainable Chem. Eng..

[cit60] Shigezawa N., Ito F., Murakami Y., Yamanaka S., Morikawa H. (2015). Development of combination textile of thin and thick fiber for fog collection bioinspired by *Burkheya purpurea*. J. Text. Inst..

[cit61] Azad M. A. K., Krause T., Danter L., Baars A., Koch K., Barthlott W. (2017). Fog Collection on Polyethylene Terephthalate (PET) Fibers: Influence of Cross Section and Surface Structure. Langmuir.

[cit62] Ganesh V. A., Ranganath A. S., Baji A., Raut H. K., Sahay R., Ramakrishna S. (2017). Hierarchical Structured Electrospun Nanofibers for Improved Fog Harvesting Applications. Macromol. Mater. Eng..

[cit63] Szewczyk P. K., Knapczyk-Korczak J., Ura D. P., Metwally S., Gruszczyński A., Stachewicz U. (2018). Biomimicking wetting properties of spider web from *Linothele megatheloides* with electrospun fibers. Mater. Lett..

[cit64] Ma M., Gupta M., Li Z., Zhai L., Gleason K. K., Cohen R. E., Rubner M. F., Rutledge G. C. (2007). Decorated electrospun fibers exhibiting superhydrophobicity. Adv. Mater..

